# Color vision in ADHD: Part 2 - Does Attention influence Color Perception?

**DOI:** 10.1186/1744-9081-10-39

**Published:** 2014-10-24

**Authors:** Soyeon Kim, Mohamed Al-Haj, Stuart Fuller, Samantha Chen, Umesh Jain, Marisa Carrasco, Rosemary Tannock

**Affiliations:** Department of Applied Psychology & Human Development, OISE, University of Toronto, 252 Bloor Street West, Toronto, M5S 1V6 Ontario Canada; Department of Psychology, York University, Toronto, Canada; Department of Psychology and Neural Science, New York University, New York, USA; Department of Clinical Psychology, University of Western Ontario, London, Canada; Department of Psychiatry, Centre for Addiction and Mental Health, Toronto, Canada

**Keywords:** ADHD, Color saturation, Contrast sensitivity, Exogenous covert attention

## Abstract

**Background:**

To investigate the impact of exogenous covert attention on chromatic (blue and red) and achromatic visual perception in adults with and without Attention Deficit Hyperactivity Disorder (ADHD). Exogenous covert attention, which is a transient, automatic, stimulus-driven form of attention, is a key mechanism for selecting relevant information in visual arrays.

**Methods:**

30 adults diagnosed with ADHD and 30 healthy adults, matched on age and gender, performed a psychophysical task designed to measure the effects of exogenous covert attention on perceived color saturation (blue, red) and contrast sensitivity.

**Results:**

The effects of exogenous covert attention on perceived blue and red saturation levels and contrast sensitivity were similar in both groups, with no differences between males and females. Specifically, exogenous covert attention enhanced the perception of blue saturation and contrast sensitivity, but it had no effect on the perception of red saturation.

**Conclusion:**

The findings suggest that exogenous covert attention is intact in adults with ADHD and does not account for the observed impairments in the perception of chromatic (blue and red) saturation.

**Electronic supplementary material:**

The online version of this article (doi:10.1186/1744-9081-10-39) contains supplementary material, which is available to authorized users.

## Background

Attention is known to compensate for the finite neural capacity in visual information processing by selecting relevant information [1–3]. Evidence from numerous neuroimaging studies support this compensatory role of attention in vision [4–7]. In particular, covert attention is considered to be a primary component of selective attention that allows us to select relevant information among competing visual stimuli without shifting one’s gaze (i.e., without saccadic eye movement) towards the relevant location [8]. There are two modes of covert attention: endogenous and exogenous attention. Endogenous attention is a voluntary system involving the dorsal frontoparietal network whereby individuals intentionally direct attention to a given location [1–3, 7–12]. By contrast, exogenous attention is an involuntary attentional system that corresponds to an automatic orienting response to a location where sudden stimulation has occurred [1–3, 6–13], and is thought to operate via a ventral frontoparietal network that is largely lateralized to the right hemisphere [10, 14].

In individuals with ADHD, findings on exogenous covert attention are still unclear. A meta-analysis on covert visuospatial attention in ADHD found only a small effect size, which was not of large enough magnitude to have clinical significance [15]. In another meta-analysis the authors concluded that exogenous covert attention is intact in ADHD [16]. On the other hand, recently, Ortega et al. [17], using ERP methodology reported that individuals with ADHD showed poorer exogenous orienting (marked by larger cue-elicited P2 activation). However, they interpreted that this result may be related to impairments in the preparation stage for target processing rather than from impairments in exogenous covert attention. Specifically, participants with ADHD exhibited reduced contingent negative variation (CNV) in the preparation stage which is considered an index of low cortical arousal, related to working memory load.

Collectively, the preceding findings suggest that visual perception and its regulation by attentional processes, particularly exogenous covert attention, warrants further investigation in ADHD. In our companion paper, we found that adults with ADHD had difficulty in color saturation discrimination (blue and red), compared to the control group, but that the two groups did not differ in contrast perception. This finding is in line with previous studies that suggested functional problems in color perception problems, particularly in blue in children with ADHD [18–21]. Here, we investigate whether exogenous attention enhances or impedes chromatic and achromatic perception in the same sample of adults with and without ADHD.

Whether and how attention affects appearance is an issue that scientists have only recently begun to investigate systematically. This may be due to the difficulty in objectively testing and quantifying the subjective experience of perceived stimuli and changes in such experience with attention. Recently, a psychophysical paradigm has been developed to assess the phenomenological correlate of involuntary attention [22], making it possible to study subjective experience more objectively and rigorously [23–26].

Among the studies that have investigated the influence of exogenous covert attention on visual perception, much work has been done examining the effects on contrast sensitivity [1, 6–8, 12, 22, 27–30]. However, to our knowledge, only one study has examined the effects of exogenous covert attention on color saturation: it found that exogenous covert attention enhanced the perception of color saturation on red, green and blue [31].

To measure the role of exogenous covert attention on color saturation and contrast sensitivity, we employed the same paradigm [22, 28–32] described in our companion paper. The key experimental manipulation was the use of different pre-cues, which appear in central or peripheral (left vs. right) locations. The pre-cue is followed by the brief presentation of two stimuli simultaneously to either side of a central fixation point: one stimulus – the ‘standard’ - has a constant saturation/contrast level, whereas the other – the ‘test’ stimulus- varies in its saturation levels or contrast levels. The fast presentation of the cue (67 ms) and stimuli (40 ms) precludes saccadic eye movement, permitting a measure of exogenous covert attention. Three cue conditions are presented in this paradigm: 1) a test cue condition in which the cue appears adjacent to the test-stimulus location; 2) a neutral cue condition in which the cue is presented at the fixation point (at the center); and 3) a standard cue condition in which the cue appears adjacent to the standard-stimulus location. The key variable of interest is the Point of Subjective Equality (PSE) in the test, neutral and standard cue conditions for each stimulus (blue, red, and contrast). The PSE is measured when the two stimuli (test and standard) look subjectively the same, and so an observer would choose randomly between them. As such, the PSE is the 0.5 probability point.

The probability of choosing the test stimuli as a function of test stimuli saturation was plotted for each cue condition using a Weibull function [33]. The PSE for each cue condition was calculated by finding the test stimulus saturation at which participants had a 50% chance of choosing the test stimuli. If exogenous covert attention enhances apparent color saturation and contrast sensitivity, pairwise comparisons should reveal significant differences between each cue type, with the PSE for the test cue condition being lower than the neutral cue, and the standard cue being higher than the neutral cue (i.e. test cue < neutral cue < standard cue). If the ADHD and control groups differed in exogenous covert attention, we would expect to find a main effect of group in PSE with different psychometric functions for PSE in the three cue conditions. If exogenous covert attention had differential effects in ADHD and control participants as a function of stimulus characteristics (e.g., chromatic versus achromatic), this would manifest as a significant interaction between group, cue, and stimulus type. Differences in PSE values among the cue conditions measure the impact of attention, independently from the saturation discrimination accuracy.

To provide a rigorous test of possible differential effects of exogenous covert attention on chromatic and achromatic stimuli, we modified parameters of the stimuli with specific characteristics that would allow us to measure different attentional influences on the three stimuli (blue, red, and contrast; see Table 1 of the companion paper for details).

**Table 1:** **Means and standard deviations in PSE as a function of cue type between ADHD and control**

	***Control***		***ADHD***		***Group***	***Cue type***	***Group*** × ***Cue type***
	***(N = 29)***		***(N = 29)***				
	***M***	***SD***	***M***	***SD***	***F***	***F***	***F***
Blue					.9	24.06**	2.04
Test	1.3206	.1718	1.2659	.2110			
Neutral	1.4505	.0743	1.4828	.0680			
Standard	1.5722	.1721	1.6403	.1708			
Red					13.16**	4.93*	.36
Test	.3570	.0154	.3614	.0134			
Neutral	.3530	.0046	.3556	.0036			
Standard	.3497	.01572	.3514	.0144			
Contrast					1.42	10**	1.57
Test	-.5700	.0811	-.6278	.2144			
Neutral	-.5123	.0182	-.5209	.02652			
Standard	-.4849	.0579	-.4577	.0882			

*p < .05; ** *p* < .001.

## Methods

### Participants

Data were derived from the same group of adults with and without ADHD as reported in Part 1 of this study (Please refer to Part 1 for a detailed description of the inclusion and exclusion criteria). Thirty subjects with a confirmed diagnosis of ADHD (50% male; age range: 18–35 years old; mean age = 24 years old) were recruited through college and university accessibility services across the Greater Toronto Area. Participants were excluded if they were currently taking sedative or mood altering medication, or stimulant medication for ADHD, and if there were any known genetic or current vision problems present in first degree family members. Thirty control subjects matched on age and gender participated. The participation was voluntary and all participants provided informed written consent before starting the study. The study was approved by the Institutional Social Sciences, Humanities, and Education Research Ethics Board (Protocol reference # 28804).

### Color saturation/contrast appearance task

The discrimination task used in our study was adapted from that implemented by Carrasco and colleagues to measure the role of exogenous covert attention in contrast sensitivity [22, 28–30, 32] and color saturation [31]. Specific modifications included: (1) the use of ‘pure’ blue and red colored stimuli to better isolate the S – (L + M) and L-M opponent cone systems, (2) constant luminance (similar to ‘brightness’) was maintained across cue and stimulus conditions, as well as between the stimuli and background; and (3) the use of the same cue size and shape across cue and stimulus conditions. These modifications permitted a more rigorous comparison of group differences in discrimination ability by ensuring that all other features of the task and stimuli remained constant across all three tasks to eliminate possible confounds. A standard set of instructions was given verbatim to participants to avoid any confounds due to misinterpretation of the instructions (Additional file 1).

### Procedures

**Figure 1:**
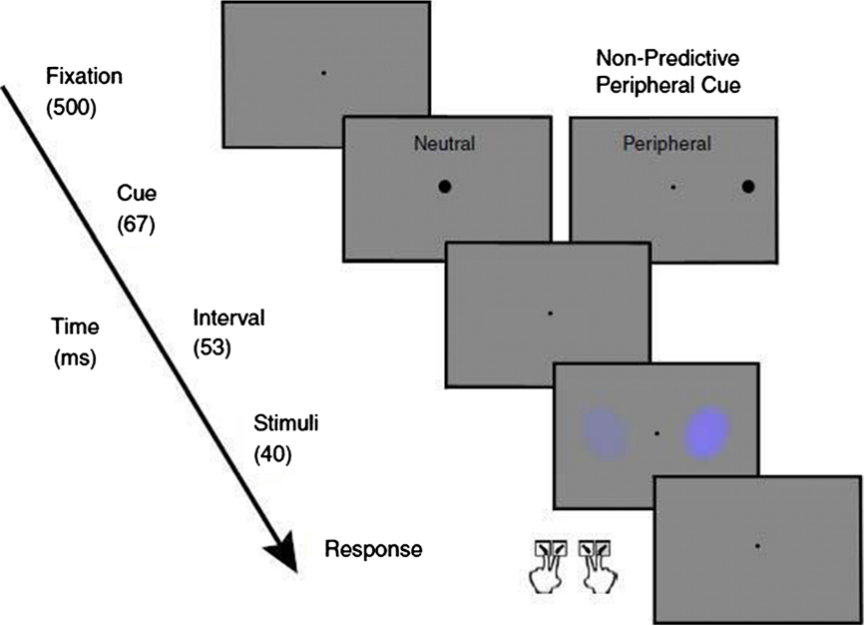
**Appearance task trial sequence.** A central fixation (500 ms) was followed by a cue (neutral or peripheral; this study includes both neutral and peripheral cue conditions). After a delay of 53 ms, stimuli were presented for 40 ms. The short period of stimuli presentation precludes saccadic eye movement, which allows for the influence of exogenous covert attention to be assessed. In each trial, participants were instructed to answer the question, "Is the stimulus that looks higher in contrast tilted to the right or left?" or "Is the stimulus that looks more colorful tilted to the right or left?" Participants chose from four options and responded by pressing one of the four designated response keys: left stimulus, tilted to left (‘z’ key); left stimulus tilted to right (‘x’ key); right stimulus, tilted to left (‘n’ key); or right stimulus, tilted to right (‘m’ key). Note that with one key press we got the orientation response, as well as the perceived saturation/contrast (the variable of more interest).

The task schematic is shown in Figure 1. In this task, participants were instructed to keep their eyes focused on a central fixation point for 500 ms. Then a cue appeared for 67 ms. in the central (neutral) or a peripheral location The peripheral cues were used to manipulate exogenous attention, and the neutral cue condition served as a baseline with which to compare the effect of attention on perception. For each trial, participants were asked to indicate whether the stimulus that looked ‘more colorful’ (or higher in contrast) was tilted to the right or the left. Participants were instructed to respond by pressing one of the four designated response keys: left stimulus, tilted to left (‘z’ key); left stimulus, tilted to right (‘x’ key); right stimulus, tilted to left (‘n’ key); or right stimulus, tilted to right (‘m’ key). To allow familiarity and proficiency with the task, participants completed 80 practice trials before beginning the actual task. Feedback response accuracy in perceived saturation/contrast and orientation accuracy (defined as selecting the correct response key for higher color saturation and orientation, or higher contrast and orientation) were shown at the end of the practice trials to ensure that the participants understood the task. Participants were required to meet cut off scores of at least 80% correct in both tasks before they could continue with the actual experimental tasks. Practice trials were not included in data analysis. Participants then completed two sessions, which were conducted on two different days. In each session, participants completed a total of 10 blocks of trials (1056 trials in total) for each color (blue, red) and for contrast, yielding a total 2112 trials for 2 sessions per task. Stimuli conditions (red, blue and contrast) were randomized. Within each stimuli condition, the cue condition (peripheral, neutral) were also randomized. Each cue condition (i.e. test, neutral, and standard) consisted of a total of 704 trials over both days. Participants were encouraged to take a short break (generally 3 minutes in length) after finishing each block. Given the high test-retest reliability (reported in the companion paper), data from the two sessions were aggregated.

### Stimuli

As described in Part 1, the color stimuli consisted of a uniformly-colored (blue or red) patch modulated by a modified Gaussian envelope that was elongated vertically and clipped at half-height. Two types of stimuli (standard and test) were presented randomly on the left and right sides of the screen. A standard stimulus has a constant saturation/contrast sensitivity, while test stimuli varied in the level of color saturation and contrast sensitivity. Standard blue stimuli had fixed color saturation (DKL saturation 1.40) whereas the saturation level of the blue test stimuli varied among 11 levels (.50 to 2.30). Similarly, the standard red stimuli had fixed color saturation (DKL saturation 0.35) and the red test stimuli varied among 11 levels (0.25 to 0.45).

For contrast stimuli, a standard Gabor (without a clipped Gaussian envelope) was presented at 3 cycles per degree (cpd) spatial frequency (approximately .36 of the total size in degrees). The standard stimuli had a fixed contrast level (28.2%), whereas the contrast level of the test stimuli varied among 11 levels (10% to 79.43%).

For all tasks (blue, red, contrast), the side of the monitor that the stimuli were presented on (right or left) and the level of the test stimuli were counterbalanced. The size of each stimulus was .36 (horizontal) × .57 (vertical) × 3° and located at 4°eccentricity. Stimuli were tilted either 20° to the left or the right. The fixation point was a 0.15° black dot. The cue was a 0.4° black dot (100 cd/ m2) located at 2° eccentricity above the stimuli. A total of 2112 trials were collected for each task which allowed for 192 trial points per each saturation level. Each cue condition (test, neutral, and standard) consisted of a total of 704 trials. For a more detailed description of the stimuli and task, see Carrasco, Ling and Read [22] for contrast and Fuller and Carrasco [31] for saturation, as well as the Appendix one of the companion paper.

### Apparatus

The stimuli, which were generated using Matlab (MathWorks, Natick, MA) and custom code, were displayed on a 21-in. Dell LCD monitor (1024 " 768 pixels at 75 Hz) with Asus 64 bit operating system. The monitor was calibrated using a Photo research PR650 SpectraColorimeter (Chatsworth, CA) and Matlab calibration routines from the PsychToolbox3.

### Analysis

Data from each individual participant were reviewed using set criteria to assess their robustness. These criteria included: 1) data points with SD's greater than 3 were regarded as outliers and adjusted using a winsorizing technique [34]; 2) participant data points with a dynamic range of the psychometric function covering less than .70 were excluded, and 3) participant data with tilt accuracy less than .80 were excluded. The dynamic range of the psychometric function and the tilt accuracy were regarded as indices of participants’ motivation level as well as their basic ability to discern different saturation levels. In the present study, we used PSE data for all three cue conditions (test, neutral and standard) to measure the impact of exogenous covert attention in the perception of color saturation and contrast sensitivity.

The main analysis compared the effects of exogenous attention on the perception of chromatic saturation and contrast sensitivity. We conducted 3 repeated measures ANOVAs separately for chromatic and achromatic stimuli conditions. Specifically, we ran 2 (group: ADHD vs. control) × 3 (cue type: test, neutral, standard) repeated measures ANOVAs using PSE values from the blue, red, and contrast discrimination tasks. If indeed the attentional pre-cue alters perception of the stimuli, then a main effect of cue type would be expected. Furthermore, post-hoc pairwise comparisons should reveal significant differences between each cue type, with the PSE for the test cue condition being lower than the neutral cue, and the standard cue being higher than the neutral cue (i.e., test cue < neutral cue < standard cue). If the ability to engage in exogenous covert attention differs between the ADHD and the control group, then a main effect of group would be expected. Specifically, if individuals with ADHD were highly susceptible to exogenous covert attention, then the effects of attentional manipulation would be greater than the control group. When viewing the psychometric functions, we would expect the distance of PSE values between the neutral cue condition and the test and standard cue conditions to be exaggerated in the ADHD group (ADHD > control). Alternatively, if exogenous covert attention was impaired in ADHD, we would expect the psychometric functions of PSE values for all cue conditions to be close to each other, showing that the attentional modulation did not have an effect on visual perception.

Given that we found sex differences in color perception in our companion paper, we conducted a supplementary analysis to investigate possible sex differences in the role of exogenous covert attention in visual perception with three separate 2 (sex: male, female) × 2 (group: ADHD, control) × 3 (cue type: test, neutral, standard) repeated measures ANOVAs for blue, red, and contrast stimuli.

Cohen’s *d* was reported to measure the standardized magnitude of group differences, which is relatively insensitive to sample size. Conventionally, Cohen’s *d* ranging from 0.2-0.3 is considered to be a small effect size, and a Cohen’s *d* of 0.5 and 0.8 are considered to be medium and large effect sizes, respectively.

## Results

1. The role of exogenous covert attention on color and contrast appearance.

**Figure 2:**
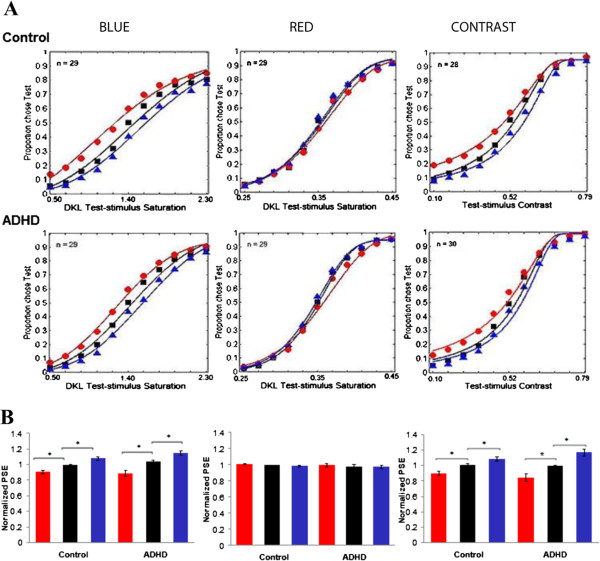
**PSE graphs. A)** Psychometric functions of color saturation and contrast sensitivity. Data from participants are combined with Weibull functions for each group. Data points are marked with symbols and fitted functions with lines. Each column presents each stimulus (blue, red, contrast). Top row presents psychometric functions for control group, and bottom row for the ADHD group. Horizontal axes represent test stimulus saturation/contrast sensitivity distances in DKL colorspace. Vertical axes are percent of trials for which the test stimulus was selected as more colourful (higher saturation level). Test cued conditions are represented by red lines with circles, neutral cued conditions by black lines with squares, and standard cued conditions by blue lines with triangles. Points of Subjective Equality (PSEs) for the Test shifts to the left for Blue and Contrast indicating that exogenous attention enhanced subjective saturation/contrast sensitivity to be more saturated or higher in contrast than the standard stimuli. Red psychometric functions indicate lack of exogenous attention influence on the stimuli as Test, and Standard psychometric functions are together with Neutral. **B)** Normalized PSE for color saturation and contrast sensitivity shown in a bar graph. Each participant’s PSEs were normalized by taking the ratio of any one PSE and the average of the test-cued, neutral, and standard-cued PSEs. Each column presents each stimulus (blue, red, contrast). In each bar graph, test cued conditions are represented by a red bar, neutral cued conditions are represented by a black bar, and standard cued conditions are represented by a blue bar. For blue and contrast, when the test stimuli were cued, participants chose the test stimuli when their saturation/contrast levels were lower than those of standard stimuli. This pattern of results indicates that cuing a stimulus increased its perceived blue saturation/ contrast sensitivity level.

As shown in Table 1 and Figure 2, a significant main effect of cue type was found for PSE for blue color saturation [*p* < .0001, *ES* = .47]. Post hoc pairwise analyses revealed significant differences between each cue type (test < neutral < standard; *p* < .001) indicating that exogenous covert attention significantly enhanced blue saturation for both groups. No main effect of group or Group × Cue Type interaction was present.

A significant main of effect of cue type was also found for red PSE [*p* = .01, *ES* = .15], but the differences in cue type were subtle in that the mean values for cue type varied over a small range (.351-.359). Post hoc pairwise comparisons revealed no significant differences between each cue type (test = neutral = standard) indicating that exogenous covert attention did not have any significant impact on the perception of red saturation, as is evident in Figure 2. No significant Group × Cue Type interaction was present. A main effect of group was present for red PSE across all cue types [*p* = .001, *ES* = .19; ADHD > control]. However, a post-hoc analysis performed to test a predicted group difference in the neutral cue condition (based on the results of the companion paper) revealed that this group difference was only present in the neutral cue condition (*p* = .019), but not in standard (*p* = .319) or test cue conditions (*p* = .375). We assume that this statistical group difference may have resulted from a very small standard deviation value in the neutral cue condition.

A significant main of effect of cue type was found for contrast PSE [*p* = .0001, *ES* = .27], as can be seen in Figure 2. Post hoc pairwise analyses revealed significant differences between each cue type (test < neutral < standard; *p* < .05) irrespective of group indicating that exogenous covert attention significantly enhanced contrast perception for both groups. No main effect of group or Group × Cue Type interaction was present for contrast PSE.

2. Sex differences in exogenous covert attention.

As seen in Table 2, we found a significant main effect of sex for blue [*F* (1, 54) = 5.17, *p* = .03, *ES* = .09; female > male]. Post-hoc analyses were performed to test a predicted sex difference in the neutral cue condition as indicated in the companion paper which indeed revealed that this sex difference was only present in the neutral cue condition (*p* = .035), whereas no differences were found in the other cue conditions. As expected from our findings in Part 1, we found a significant main of effect of cue type for blue PSE. A significant main of effect of cue type and group was also found for red PSE (as described in the previous section of the current results), and there was a significant main effect of cue type for contrast (as described in the previous section of the current results).

**Table 2:** **Means and standard deviations in PSE as a function of cue type between male and female participants**

	Male				Female									
	Control (***n*** = 15)		ADHD (***n*** = 13)		Control (***n*** = 14)		ADHD (***n*** = 16)		Cue type ( ***F*** )	Sex ( ***F*** )	Group ( ***F*** )	Cue type × Sex ( ***F*** )	Cue type × Group ( ***F*** )	Cue type × Sex × Group ( ***F*** )
	***M***	***SD***	***M***	***SD***	***M***	***SD***	***M***	***SD***						
Blue									23.18**	5.17*	.80	.08	2.00	.50
Test	1.30	.16	1.25	.21	1.35	.18	1.28	.22						
Neutral	1.42	.08	1.48	.05	1.49	.05	1.48	.08						
Standard	1.54	.17	1.64	.16	1.60	.17	1.64	.18						
Red									4.67*	.16	13.07**	.01	.36	.35
Test	.36	.02	.36	.01	.36	.02	.36	.01						
Neutral	.35	.00	.36	.00	.35	.00	.36	.00						
Standard	.35	.02	.36	.01	.35	.01	.35	.01						
Contrast									9.74**	1.05	1.29	.54	1.54	.54
Test	-.55	.07	-.60	.15	-.59	.09	-.65	.26						
Neutral	-.51	.02	-.52	.02	-.52	.02	-.52	.03						
Standard	-.50	.06	-.46	.09	-.47	.06	-.45	.09						

*p < .05; ** *p* < .001.

## Discussion

The present study is the first to examine the effects of exogenous covert attention on chromatic and achromatic perception in individuals with ADHD. The main finding was that the effects of exogenous covert attention on chromatic (blue and red) as well as achromatic perception were similar for both the ADHD and control groups. Specifically, attention significantly enhanced perceived blue saturation and perceived contrast but did not influence the appearance of red saturation in either the ADHD or control groups. Moreover, there was no evidence that exogenous attention was altered in ADHD, suggesting that this form of selective attention is intact in ADHD.

The lack of a group difference in the exogenous covert attention towards chromatic and achromatic perception rules out the possibility that exogenous covert attention may account for the deficiency in chromatic saturation discrimination in adults with ADHD, as reported in our companion paper.

Furthermore, we found no evidence that exogenous covert attention was altered in ADHD, suggesting that it is intact in ADHD. This finding adds to a growing literature which suggests that exogenous covert attention may be spared in ADHD. This result is in line with previous studies including the meta-analysis on covert attention in ADHD [15, 35]. Specifically, in a meta-analysis on covert orienting in individuals with ADHD, Huang-Pollock and Nigg [15] found that the effect sizes for group differences were homogeneously small, which parsimoniously prohibit concluding that covert attention/orienting is impaired in ADHD population. In another meta-analysis based on studies that examined serial visual search tasks, Mullane and Klein [16] also concluded that exogenous covert attention is intact in children with ADHD. Endogenous visual attention, however, yielded a variable result and was concluded to be less efficient in individuals with ADHD, which is in line with findings from Wood et al. [36]. However, the preceding findings regarding intact exogenous covert attention in ADHD are inconsistent with a MEG/ERP study which showed reduced activation in the ventral attentional pathway [37], which is suggested to mediate exogenous attention. Perhaps using a Go-NoGo task was not a direct measure for exogenous attention as attention is not explicitly manipulated in this paradigm, and is commonly used to test inhibition in ADHD population and other special populations. The lack of attentional influence on red perception in the control group is inconsistent with a previous study [31]. Possibly, methodological differences (summarized in Table 3), may account for this inconsistent result. The difference in ‘purity’ in red stimuli was explored, where neither pure nor impure red stimuli showed effects on exogenous covert attention (Additional file 2). Whether attention influences the perception of red color is inconclusive. Further study is needed to confirm an effect of attention on red perception that will require a more comprehensive investigation of all parameters, one at a time and in combination.

**Table 3:** **Comparison of parameter variables**

Parameter		Fuller et al. 2006 [31]	Current study
**Color stimuli purity** (in DKL space)	S-Cone	On S cone axis	On S cone axis
	LM-Cone	On a diagonal between LM and S cone axes	On LM cone axis
**Saturation level (=11)**	S-Cone	1.52 ~ 4.70 (Standard = 2.87)	0.50 ~ 2.30 (Standard = 1.40)
	LM-Cone	0.81 ~ 1.07 (Standard = 0.95)	0.25 ~ 0.45 (Standard = 0.35)
**Luminance**	S-Cone	15 candella/m^2^	30 candella/m^2^
	LM-Cone	20 candella/m^2^	30 candella/m^2^
	Background	3 candella/m^2^	30 candella/m^2^
**Cue**		White	Black
**Cue shape & size**		Round, .3 × .3	Round, .4 × .4

To our knowledge, no previous study has investigated sex differences in exogenous covert attention in ADHD. Our results suggest that exogenous covert attention may work similarly for both males and females with and without ADHD. With healthy adults, Bayliss and colleagues [38] found that male and female attentional systems treat exogenous cues in similar ways. Studies that investigated endogenously cued attention such as vigilance and selective attention showed differential responses between the sexes. For instance, males are suggested to have greater selective spatial attention than females (using endogenously cued paradigm), which may be attributed to better spatial abilities [39, 40].

This study has some limitations. First of all, we did not have a direct measure of IQ, or scholastic ability, which limits the characterization of our sample. However, these college students must be of at least average intelligence to be able to successfully complete high school and gain college entrance. Furthermore, although we excluded all participants who reported any genetic visual problems as well as any visual problems in their families, our study could not empirically verify the presence of heterozygosity in X-linked cone photopigment expression or the nature of it (protan vs. deutan) in female participants. 15% of females are reported to inherit X-chromosome carrying an abnormal L/M array which could affect chromatic sensitivity [41]. Future studies should scrutinize all possibilities of genetic predisposition of chromatic perception.

## Conclusion

Despite the aforementioned limitations, in this paper, we show that exogenous covert attention does not play a role in the altered color perception in ADHD, which we reported in the companion paper. Specifically, we found that adults with ADHD made more errors than controls in discriminating higher blue and red saturation while not in contrast sensitivity, but that they did not differ from normal controls in terms of the beneficial effects of exogenous attention on the perception of the saturation of blue or on contrast sensitivity.

Future investigations examining the role of endogenous attention in visual perception should ascertain whether the differential effect of attention on red saturation is specific to exogenous covert attention. Also, using the fine temporal resolution of ERP methodology, one could measure pre-specified ERP components when exogenous covert attention is elicited and chromatic and achromatic stimulus is presented. This study may serve to find out if the involvement of the early visual cortex manifested in neurotypical observers [25, 42] would appear in individuals with ADHD as well.

## Electronic supplementary material

Additional file 1: **Instruction for the appearance task.** (DOCX 16 KB)

Additional file 2: **Small scale follow up study.** (DOCX 133 KB)
