# Supplementary material for: Color vision in ADHD: Part 2 - Does Attention influence Color Perception?
Source: Behav Brain Funct. 2014 Oct 24;10:39. doi: 10.1186/1744-9081-10-39 (PMC4282194; doi:10.1186/1744-9081-10-39)
Supplement: Supplementary file 1 — Additional file 1: Instruction for the appearance task. (DOCX 16 KB) [file 12993_2014_513_MOESM1_ESM.docx]

Appendix 1. Instruction for the appearance task

"Now, you will be looking at pairs of small stimuli, presented briefly against a gray background on a computer screen in a darkened room. For each pair, you will make a relative judgment of them to decide which of them looks more “intense”. In addition, the two stimuli will be randomly and independently tilted to the right or left. The question that you will answer is: “**in which direction (right or left) is the more ‘intense’ of the two stimuli tilted?**” You will indicate your answer by pressing a single key on the computer keyboard.

* What does "intense" mean for these stimuli?

COLORS:

[Showing the demo stimuli] you’ll see when you look at the entire range we’re using, that some are “**less colorful**” or “**washed out**”, whereas at the other end of the range they are “**more colorful**” or “**richer**” or “**purer**”, and probably easier to distinguish from the gray background.

CONTRAST:

[Showing the demo stimuli] For contrast, it basically means we want you to choose and report on the tilt of the **higher contrast** stimulus of the pair. The higher contrast stimulus has **whiter whites and blacker blacks** in it than the lower contrast stimulus, and it’s easier to distinguish from the gray background.

[Showing the task scheme]

* Throughout the experiment, there will be a black dot in the center of the screen. It’s important that you **keep your eyes focused on that dot**. A larger black dot will flash, over the smaller center dot or next to one of the locations of the two stimuli. The location of this warning dot will be random, and it has **nothing to do with the stimuli or which one is more “intense”**. Except for telling you the stimuli are about to appear, you can ignore it. After the dot, there will be a short interval, then the stimuli will appear and disappear. At this point, the computer will wait for your answer. The next trial will start only after you press a key.

*[Show the keys on the keyboard] We have set up **four active keys** on the keyboard for the experiment, two that you can operate with your left hand representing the stimulus on the left side of the screen, and two you can operate with your right hand representing the stimulus on the right side of the screen. The two keys for each side represent left tilt and right tilt. If you set up your fingers on these keys as shown in the little diagram, this should be fairly simple with a little practice.

The **training program** will give you some practice with the task, and pairs of stimuli that are pretty easy to distinguish. In the actual experiment, there will be pairs that are somewhat easier to distinguish, and some that are harder. In some cases, you may not really be sure which stimulus was more intense. In such cases, just go with your 'gut feeling', but do try to give the correct tilt for whichever one you pick.
