# Supplementary material for: Color vision in ADHD: Part 2 - Does Attention influence Color Perception?
Source: Behav Brain Funct. 2014 Oct 24;10:39. doi: 10.1186/1744-9081-10-39 (PMC4282194; doi:10.1186/1744-9081-10-39)
Supplement: Supplementary file 2 — Additional file 2: Small scale follow up study. (DOCX 133 KB) [file 12993_2014_513_MOESM2_ESM.docx]

**Appendix B. Follow up study**

**Rational**:

The follow up study was conducted to further examine the lack of exogenous covert attention on red saturation perception in healthy adults. This finding is in contrast to Fuller and Carrasco (2006) who found an effect of exogenous covert attention on red saturation perception. Thus, further investigation is required to better understand the discrepant results.

In contrast to our main study, Fuller and Carrasco (2006) did not intend to compare the effect of exogenous covert attention between colors; hence several parameters of the color stimuli were different (see Table 3). Specifically, Fuller and Carrasco (2006) used impure red stimuli. In DKL space, the impure color would be represented in the middle of the blue (S-cone) and red (LM-cone) axes. In the current follow up study, we examined whether difference in the ‘purity’ of the red stimuli had a differential effect on the results.

**Method**:

*Participants:* 9 control participants who participated in the main study were randomly selected and asked to participate in the follow up study (female = 4, mean age = 26).

*Procedure:* Participants completed a total of 10 blocks of trials (1056 trials in total) for 3 tasks (impure red, pure red and blue). Stimuli conditions (impure red, pure red and blue) were randomized. Within each stimuli condition, the cue condition (peripheral, neutral) was also randomized. The general procedure was kept the same as the main study.

*Stimuli modifications:* The pure red and blue stimuli had the exact same parameters as the main study, while the impure red task used the same DKL coordinates as in Fuller and Carrasco (2006). Except for the “purity” of the impure red stimuli, all of the other task parameters were kept constant between the tasks.


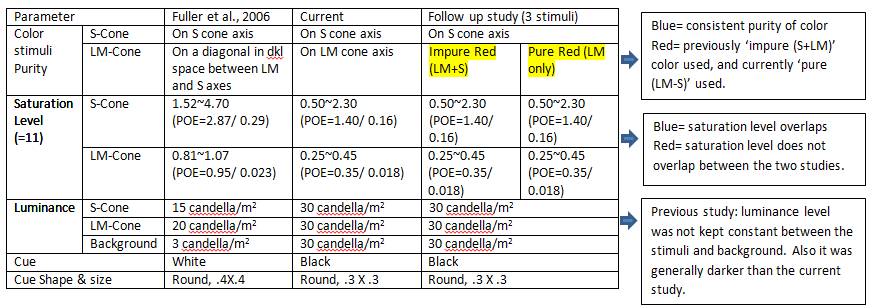


**Result:**

The follow-up study replicated findings from our main study, showing similar PSE psychometric function for pure red and blue. The test-cued psychometric function for blue shifted significantly to the left, indicating that the participants were more likely to choose the test stimulus as being of a higher saturation level when it was cued (test condition < neutral condition < standard condition). However, neither pure red nor impure red revealed similar psychometric functions as blue, indicating a lack of cue (attention) effect on both pure and impure red stimuli.


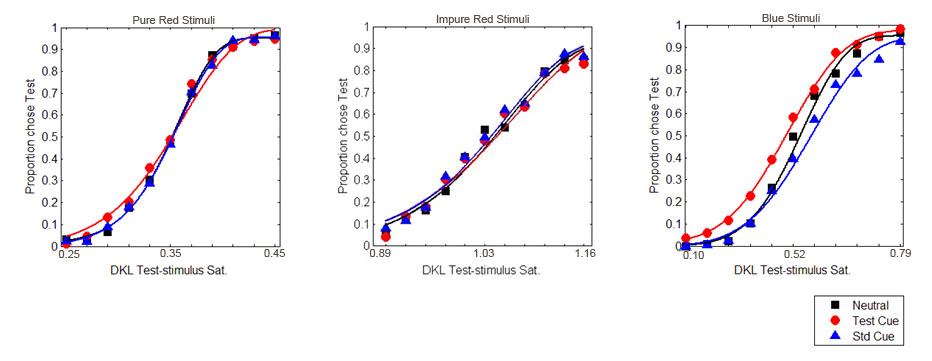


**Conclusion**:

The discrepant findings may be attributable to a more complex & specific influence between the color of the stimuli and the background luminance that holds for red, but not blue or contrast. Whether attention influences perception red saturation perception is inconclusive. Further investigations are required to confirm the lack of effect of exogenous covert attention on red saturation perception that will require a more comprehensive investigation of all parameters, one at a time and in combination.
